# The out‐of‐pocket cost of living with obesity: Results from a survey in Spain, South Korea, Brazil, India, Italy, and Japan

**DOI:** 10.1002/osp4.70000

**Published:** 2024-08-14

**Authors:** Karine Ferreira, Evant Kont, Amira Abdelkhalik, Dominic Jones, James Baker‐Knight

**Affiliations:** ^1^ Novo Nordisk A/S Søborg Denmark; ^2^ Ipsos London UK

**Keywords:** health economics, health expenditure, weight loss

## Abstract

**Objectives:**

In many countries, obesity treatments are not fully reimbursed by healthcare systems. People living with obesity (PwO) often pay out‐of‐pocket (OOP) for pharmacological and non‐pharmacological interventions, placing them in a position of financial risk to manage their condition. This study sought to understand the OOP expenditures and non‐financial costs incurred by PwO to manage weight.

**Methods:**

A 25‐min cross‐sectional online survey was conducted with PwO between ages 18–60 in Italy, Japan, India, Brazil, Spain and South Korea. Respondents were recruited using proprietary vendor panels and non‐probability sampling. *N* = 600 participants completed the survey (*n* = 100 per country).

**Results:**

The mean annual OOP expenditure related to weight loss/management was $7,351, accounting for nearly 17% of annual household income. Costs generally increased by BMI. Half or more of the respondents agreed that obesity affected multiple aspects of their lives (outside activities, running a household, social life, work, family life, traveling). 46% agreed that obesity limited their job prospects.

**Conclusion:**

PwO spend a notable amount of their income paying OOP expenditures related to managing their weight. Quantifying the individual economic burden of living with obesity can inform the understanding of the resources required and policy changes needed to treat obesity as a disease.

**Abbreviations**
BMI: body mass index
GDP: gross domestic product
IRB: institutional review board
OOPs: out‐of‐pocket payments
WHO: World Health Organization

## 1 INTRODUCTION

Out‐of‐pocket payments (OOPs) account for more than 20% of health spending on average across Organization for Economic Cooperation and Development (OECD) countries.^1^ These are defined as individuals' direct payments to healthcare professionals at the time of service use.^2^ OOP as a percentage of private expenditure on health are used as a core indicator by the World Health Organization (WHO) to assess health financing systems.^3^ High OOPs are an indicator of poor systems and are strongly associated with catastrophic health spending (where OOPs are greater than 40% of capacity for pay for healthcare at a household level, after food and housing^4^). High OOPs have also been shown to lead to poverty for socioeconomically vulnerable groups,^5^ particularly in low‐ and middle‐income countries where OOP spending is proportionally higher.^6^ Although obesity affects more than 650 million people worldwide^7^ and has been recognized by the WHO as a disease,^8^, ^9^, ^10^ in many countries healthcare decision‐makers do not classify obesity as such. As a result, obesity treatments are not reimbursed by the healthcare system, and people with obesity often must pay OOP for pharmacological and non‐pharmacological interventions,^11^ placing them in a position of financial risk to manage their condition.

Current OOP expenditures may include lifestyle interventions, which are currently the cornerstone of weight management, but people who rely on these interventions alone often struggle to maintain weight loss.^12^ Although the efficacy of anti‐obesity medications has been limited until fairly recently, pharmacotherapy (including treatments for both chronic and short‐term weight management) can also be used as an adjunct to lifestyle interventions to help individuals living with overweight and obesity achieve and sustain clinically relevant weight loss.^13^ Bariatric surgery provides greater weight loss of up to 45%^14^ but is typically reserved for patients with BMI ≥40 kg/m^2^, or BMI 35.0–39.9 kg/m^2^ with at least one weight‐related comorbidity, with further restrictions placed on reimbursement and patient access in many countries.^15^, ^16^

The continued increase of obesity will have a substantial financial impact for individuals and health systems alike: across eight countries in 2019, it was estimated that obesity cost between 0.80% and 2.42% of gross domestic product (GDP) and, if current trends continue, this will increase to an average of 3.6% of GDP by 2060.^11^ Improvements in obesity‐related comorbidities may translate into substantial healthcare cost savings.^17^, ^18^

Despite the relevance of this issue, there is limited literature capturing individual OOP expenditures on weight management.^19^ Existing literature primarily captures per capita spending and very few studies report disaggregated costs on what patients are spending money on outside the United States.^19^ Furthermore, there are scant evidence available on the economic impacts of obesity that are comparable across income contexts for policy and advocacy.^11^ Previous studies have called for a holistic assessment of the costs of treating obesity, which can provide recommendations for policy and decision‐makers to design obesity treatment programs.^19^ Understanding the OOP expenditures incurred by people with obesity is critical to understand the full economic burden of living with this disease, and to help healthcare decision‐makers make informed decisions about interventions. To this end, a cross‐sectional study was conducted to understand the individual financial OOP expenditures and perceived non‐financial costs (social, emotional, and physical) incurred by people living with obesity (PwO). Six different countries were included with differing prevalence of PwO in accordance with the World Obesity Federation: Japan (7.6% of men and 3.6% of women), Italy (17.9% of men and 17.7% of women), Spain (19.4% of men and 13.0% of women), India (5.4% of men and 9.8% of women), Brazil (25.1% of men and 32.6% of women) and South Korea (8.8% of men and 5.7% of women).^20^

## 2 METHODS

### 2.1 Study design

Pilot interviews were conducted to test whether the questionnaire was clear enough for self‐completion. The finalized survey was then scripted and administered using an online platform system.

The 25‐min quantitative survey was conducted online between 27 April and 7 July 2022, in Italy, Japan, India, Brazil, Spain, and South Korea. Participants read the privacy and consent text at the start of the interview. Respondents indicating “Yes, I consent to my personal data being collected and processed for market research and analysis purposes” participated in the survey. Those indicating no were excluded from participation.

### 2.2 Setting

Respondents in Italy, Japan, India, Brazil, Spain, and South Korea were recruited online using consumer panels which are databases built over time to include members of the public who have indicated willingness to take part in surveys through open recruitment and direct campaigns. These panels implement automated and manual controls at recruitment stage and, for each survey, to eliminate bots new members are validated through multiple processes (open questioning, check of IP addresses and digital fingerprints, requiring identified postal address in the local country and IBAN of a bank account). Respondenses are checked by the panel and those presumed to be fraudulent (e.g., bad quality answers in open‐ended questions, speeders or straight‐line responders) are removed from the panel on a regular basis.

### 2.3 Participants

#### 2.3.1 Inclusion criteria

A non‐probability sampling method was employed to identify participants with the following criteria: (1) describing themselves as overweight or obese, (2) a BMI ≥25 by self‐reported height and weight, (3) if defined as overweight had at least one comorbidity, (4) between ages 18–60 (up to 64 in Japan), and (5) willing to personally pay for a physician‐prescribed medication for weight loss/weight management. BMI was calculated based on the respondent's self‐reported weight and height and varied by country; ranges were used based on World Obesity Federation classifications^21^ and respect the specific medical country definition for each subgroup.

#### 2.3.2 Exclusion criteria

Exclusion criteria included: (1) being diagnosed with diabetes (Type I, Type II) by a health care provider, (2) having never previously spoken to a healthcare provider about weight loss and having never attempted to make lifestyle changes to reduce weight, (3) reporting being “not at all willing” to pay for OOP medication, and (4) living in a rural/countryside area. These exclusion criteria were applied to (1) ensure the research was focused on those diagnosed with obesity and not confounded by another comorbidity like diabetes, (2) include those who would be willing to attempt to lose weight, (3) ensure respondents had some level of willingness to pay for anti‐obesity medication, and (4) ensure participants had access to weight loss and lifestyle modifications. This was determined by indicating “very willing,” “fairly willing,” or “not very willing” to the question “to what extent would you be willing or not to personally pay for a physician‐prescribed medication for weight loss/weight management, if at all?”

### 2.4 Variables

*Income*: Respondents self‐reported their monthly household income and were then classified into income groups per country (Table A1) using data on average income from publicly available sources (e.g., World Bank) and approved by local stakeholders in each country.

**FIGURE 1:**
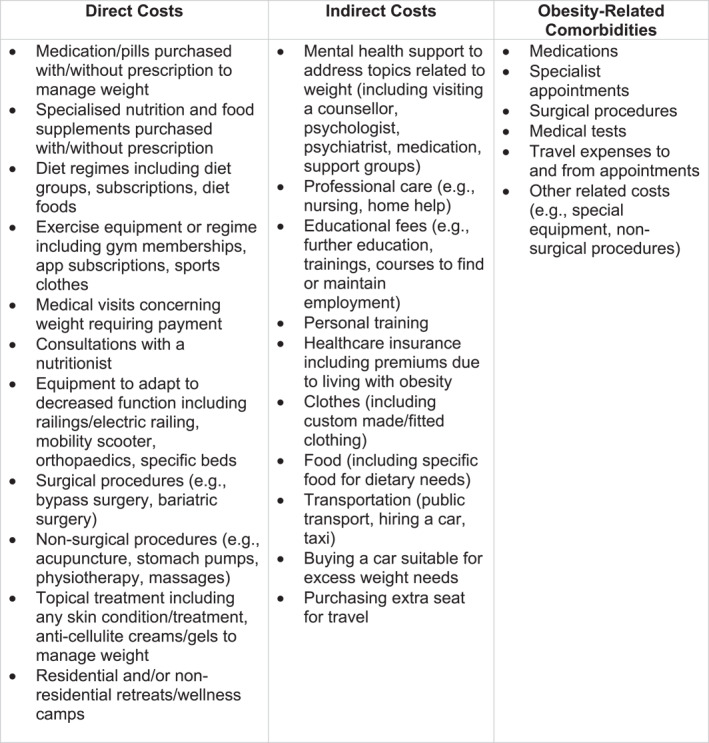
Out‐of‐pocket cost categories and details.

*Out‐of‐pocket cost categories*: Figure 1 provides specific details of costs collected in each category; broadly, direct costs were defined as costs directly related to weight loss and/or management (e.g., diet food, medication, exercise equipment, medical visits related to weight), indirect costs were those spent related to or as a result of living with obesity (e.g., cost of mental health support related to living with obesity, cost of home help or professional care needed as a result of living with obesity). Like direct costs, obesity‐related comorbidity costs captured included medication, tests, and consultation fees. All costs were converted from local currency into real 2021 United States Dollars (USD) using the following exchange rates: Spain, 1 EUR = 0.96 USD; Brazil, 1 BRL = 0.175 USD; Italy, 1 EUR = 0.986 USD; India, 1 INR = 0.018 USD; Japan, 1 JPY = 0.007 USD; South Korea, 1 KRW = 0.0007 USD. Definitions for terms were provided in the survey, including for medication (any medicines or pills you buy or get with or without a prescription to manage your weight); food (specific food for dietary needs); specialized nutrition and food supplements (any special treatments apart from medicines or pills, usually wet or dry blends, vitamins you buy with or without a prescription to manage your weight); diet regimes: (specific diet groups such as Weight Watchers where you subscribe and purchase special foods or following a specific diet such as low/no carb); and exercise and fitness (exercise equipment, places where you exercise or exercise regimes, including equipment, gym membership, app subscriptions, sports clothes).

### 2.5 Data sources/measurement

Survey questions were closed‐ended, with Likert scales used for attitudinal measurement for some questions or single‐select responses from a list of pre‐determined responses. Participants were asked about costs incurred related to managing weight and weight loss, including direct costs, indirect costs, and obesity‐related comorbidities treatments. For each cost reported, participants were then asked to estimate costs spent on each item over different time periods (e.g., last three, six or 12 months; ever), and estimate total expenditure for one‐time purchases (e.g., car, surgical procedure). Participants were also asked about their attitudes toward expenses paid related to weight loss/management and the impact of living with obesity on various aspects of their lives. All interview responses were coded directly into an online script during the interview and then uploaded to a server at the end of the interview. Respondents were allowed to skip any questions they preferred not to answer. If questions were skipped, data were coded as “prefer not to answer” and included in final tabulations of the data. Data were checked on a rolling basis for missing responses and/or unusual response patterns. Only the completed questionnaires were analyzed.

### 2.6 Study size

Total *N* = 600 participants (*N* = 100 participants per country) participated in the research. Details on those who were contacted but did not participate/screen‐out details were not maintained by the fieldwork provider. No power calculation was conducted to achieve the desired sample as no a priori confidence intervals were set prior to conducting the study, and the aim of the analysis was to be descriptive. Instead, it was determined that a sample of *N* = 100 would be recruited in each country.

### 2.7 Analysis

Costs were calculated per category using mean and median figures. Mean expenditure per item/cost category represents the average expenditure across all respondents and was calculated by summing all expenditure values for a given item/category and dividing by the number of respondents who purchased that item/category. Median expenditure represents the expenditure of a typical respondent on an item/cost category and was calculated by ordering expenditure values from least to greatest and finding the middle value. The total overall results are based on country averages across the six countries and each country was weighted equally.

Data shown in this manuscript were analyzed using descriptive statistics (e.g., mean, frequency). In some cases, Top 2 Box results (T2B; e.g., points 1 and 2 on a 5‐point Likert scale) or Bottom 2 Box (B2B; e.g., points 4 and 5 on a 5‐point Likert scale) were presented.

### 2.8 Ethics

The study was conducted in accordance with the Declaration of Helsinki. The research protocol and materials were submitted, reviewed, and approved by an independent institutional review board (IRB). The IRB was transparent in its functioning, independent of the researcher, the sponsor, and any undue influence, and duly qualified. The research was determined to be Exempt according to FDA 21 CFR 56.104 and 45CFR46.104(d) (2): (2) Tests, Surveys, and Interviews on 1 April 2023 by Pearl IRB.

## 3 RESULTS

Of the final sample (*n* = 600), 55% of respondents were male and 71% were age 36 or older. Respondents were distributed across BMI class: 22% were classified as overweight, 31% in BMI Class 1, 26% in BMI Class 2, and 21% in BMI Class 3. 44% of respondents were classified as low income compared to 34% medium and 22% high income; additional demographic details are in Table 1.

**TABLE 1:** Final sample demographics.

	Total	Brazil	Spain	India	Japan	South Korea	India
Total	600	100	100	100	100	100	100
Gender							
Male	328 (55%)	42 (42%)	27 (27%)	54 (54%)	79 (79%)	77 (77%)	49 (49%)
Female	272 (45%)	58 (58%)	73 (73%)	46 (46%)	21 (21%)	23 (23%)	51 (51%)
Age							
18–35	172 (29%)	45 (45%)	20 (20%)	42 (42%)	29 (29%)	17 (17%)	19 (19%)
36+	428 (71%)	55 (55%)	80 (80%)	58 (58%)	71 (71%)	83 (83%)	81 (81%)
Weight							
Overweight	130 (22%)	28 (28%)	24 (24%)	22 (22%)	0 (0%)	29 (29%)	27 (27%)
BMI class 1	187 (31%)	37 (37%)	27 (27%)	30 (30%)	38 (38%)	29 (29%)	26 (26%)
BMI class 2	156 (26%)	21 (21%)	24 (24%)	24 (24%)	28 (28%)	26 (26%)	23 (23%)
BMI class 3	127 (21%)	14 (14%)	25 (25%)	24 (24%)	24 (24%)	16 (16%)	24 (24%)
Income							
Low	261 (44%)	66 (66%)	50 (50%)	19 (19%)	54 (54%)	38 (38%)	34 (34%)
Medium	206 (34%)	26 (26%)	33 (33%)	38 (38%)	21 (21%)	47 (47%)	41 (41%)
High	133 (22%)	8 (8%)	17 (17%)	43 (43%)	25 (25%)	15 (15%)	25 (25%)

Details on financial OOP expenditures (direct costs, cost of comorbidity treatment, indirect costs) are reported in Tables 2, 3, 4, 5. Table 2 shows detailed expenditures by country. The mean expenditure on weight management of any kind was $7351 per year, ranging between $3856 (Brazil) and $17,076 (Japan). Across all respondents, direct costs related to living with obesity accounted for 59% of expenditure, compared to 24% for indirect costs and 17% for costs related to comorbidities. At a country level, this trend held largely except for India where expenditure related to comorbidities was higher (23%, compared to 54% direct and 23% indirect costs); and indirect costs were higher in Japan (30%, compared to 55% direct and 16% comorbidities cost). Overall, weight management expenditure accounted for an estimated 17% of annual household income; at the country level, South Korea spent the least % of annual household income (13%), and Japan spent the most (25%). In between were Brazil and Spain (both 14%), India (15%) and Italy (23%).

**TABLE 2:** Average yearly expenditure by country, mean summary (USD).

	Total	Brazil	Spain	Italy	India	Japan	South Korea
Direct costs							
Base: All who purchase the product	*N* = 406	*N* = 83	*N* = 6	*N* = 61	*N* = 90	*N* = 46	*N* = 65
Medication	$345.20	$208.30	$282.30	$414.20	$388.10	$601.90	$373.90
Specialized nutrition + food supplements	$359.20	$218.30	$649.60	$504.20	$300.50	$547.50	$310.80
Diet regimes	$425.70	$200.70	$578.90	$386.10	$315.20	$1305.70	$319.00
Exercise gear	$451.20	$240.70	$452.10	$594.50	$419.80	$493.90	$667.00
Medical visits	$369.80	$188.70	$313.20	$422.20	$254.90	$1153.90	$104.60
Consultations with a nutritionist	$373.90	$171.10	$389.50	$425.20	$257.70	$1261.80	$159.90
Specific equipment (e.g., mobility scooter, orthopedics)	$304.30	$204.70	$227.00	$473.40	$257.00	$651.10	$293.50
Surgical procedures	$562.90	$57.00	$391.00	$529.70	$295.80	$1378.10	$209.60
Residential retreats/wellness camps	$258.20	$109.40	$73.50	$858.30	$170.00	$584.20	$201.70
Non‐residential retreats/wellness camps	$231.90	$100.50	$146.20	$239.60	$180.30	$604.40	$358.70
Non‐surgical procedures (e.g., acupuncture, stomach pumps, physiotherapy)	$433.80	$300.70	$608.80	$841.40	$360.30	$607.50	$254.30
Topical treatment (for any skin condition, e.g., anti‐cellulite gels or creams)	$239.30	$203.60	$211.10	$313.80	$259.90	$183.10	$256.60
Indirect costs							
Base: All who purchase the product	*N* = 202	*N* = 50	*N* = 20	*N* = 20	*N* = 67	*N* = 20	*N* = 25
Mental health support	$340.50	$274.80	$429.80	$601.70	$344.60	$261.80	$286.60
Professional care	$499.00	$563.50	$106.50	$497.80	$346.10	$1956.40	$160.10
Educational fees	$311.40	$240.60	$224.30	$276.00	$395.90	$241.90	$200.80
Healthcare insurance	$584.80	$0.00	$753.50	$303.60	$352.60	$2548.30	$362.40
Comorbidity costs							
Base: All who purchase the product	*N* = 388	*N* = 66	*N* = 69	*N* = 70	*N* = 61	*N* = 55	*N* = 67
Medications	$274.80	$221.80	$217.40	$385.40	$254.80	$236.70	$346.40
Specialist appointments	$396.00	$256.80	$726.20	$828.50	$271.00	$231.60	$220.10
Surgical procedures	$1506.70	$1267.00	$5972.50	$98.30	$984.20	$8320.50	$652.70
Medical tests	$475.20	$166.70	$369.00	$539.70	$294.80	$1728.90	$287.60
Travel expenses to and from appointments	$294.80	$115.10	$174.00	$180.90	$202.30	$1063.00	$110.30
Other related costs (e.g., special equipment, non‐surgical procedures)	$853.00	$109.40	$1085.80	$106.40	$596.20	$4640.40	$195.60
Total expenditure (annual)	$7351.00	$3856.40	$6840.90	$8853.80	$6393.60	$17076.00	$5324.10
Direct (%)	59	57	63	68	54	55	66
Indirect (%)	24	28	22	19	23	29	19
Comorbidities (%)	17	15	15	13	23	16	15
Average annual household income	$44,271.70	$27,285.50	$48,509.40	$38,676.00	$42,100.40	$68,872.80	$40,186.10
Proportion of annual household income spent (%)	17	14	14	23	15	25	13

**TABLE 3:** Average yearly expenditure by BMI class, mean summary (USD).

	Overweight + 1 comorbidity (Excl Japan)	BMI class 1	BMI class 2	BMI class 3
Direct costs				
Base: All who purchase the product	*N* = 89	*N* = 132	*N* = 97	*N* = 88
Medication	$271.8	$227.8	$418.9	$493.0
Specialized nutrition + food supplements	$292.0	$308.6	$534.1	$361.7
Diet regimes	$324.4	$247.6	$775.1	$499.5
Exercise gear	$645.2	$367.4	$376.9	$443.1
Medical visits	$249.7	$181.9	$695.0	$447.9
Consultations with a nutritionist	$242.3	$236.9	$557.5	$529.8
Specific equipment (e.g., mobility scooter, orthopedics)	$340.3	$180.0	$478.0	$264.1
Surgical procedures	$530.4	$161.6	$983.3	$730.4
Residential retreats/wellness camps	$288.1	$159.5	$459.6	$236.1
Non‐residential retreats/wellness camps	$207.4	$119.5	$520.1	$176.3
Non‐surgical procedures (e.g., acupuncture, stomach pumps, physiotherapy)	$546.5	$162.2	$610.7	$530.1
Topical treatment (for any skin condition, e.g., anti‐cellulite gels or creams)	$316.5	$150.2	$265.3	$240.6
Indirect costs				
Base: All who purchase the product	*N* = 47	*N* = 71	*N* = 36	*N* = 48
Mental health support	$292.1	$236.5	$536.6	$399.1
Professional care	$421.8	$261.4	$1194.7	$373.7
Educational fees	$282.5	$290.2	$484.6	$270.8
Healthcare insurance	$396.2	$383.5	$1253.8	$452.1
Comorbidity costs				
Base: All who purchase the product	*N* = 103	*N* = 121	*N* = 96	*N* = 68
Medications	$277.5	$217.5	$386.3	$258.7
Specialist appointments	$446.7	$255.2	$664.3	$372.8
Surgical procedures	$726.0	$797.1	$3390.0	$1923.3
Medical tests	$259.8	$195.0	$1355.0	$330.5
Travel expenses to and from appointments	$162.5	$100.0	$780.4	$183.3
Other related costs (e.g., special equipment, non‐surgical procedures)	$227.7	$502.7	$2567.6	$446.3
Subtotal: Direct costs	$4254.5	$2502.9	$6674.4	$4952.5
Subtotal: Indirect costs	$1392.6	$1171.6	$3469.7	$1495.6
Subtotal: Comorbidity costs	$878.6	$705.2	$2293.3	$1463.8
Total costs (annual)	$6525.7	$4379.7	$12,437	$7911.9
Average annual household income	$55,072.48	$42,110.43	$36,286.89	$42,519.47
Proportion of annual household income spent (%)	12	10	34	19

**TABLE 4:** Average yearly expenditure by gender, mean summary (USD).

	Male	Female
Direct costs		
Base: All who purchase the product	*N* = 215	*N* = 191
Medication	$361.10	$323.10
Specialized nutrition + food supplements	$327.90	$399.40
Diet regimes	$332.00	$521.60
Exercise gear	$405.00	$503.50
Medical visits	$343.60	$401.70
Consultations with a nutritionist	$347.50	$400.90
Specific equipment (e.g., mobility scooter, orthopedics)	$280.70	$339.70
Surgical procedures	$635.30	$345.80
Residential retreats/wellness camps	$174.0	$364.40
Non‐residential retreats/wellness camps	$272.90	$181.20
Non‐surgical procedures (e.g., acupuncture, stomach pumps, physiotherapy)	$386.90	$491.30
Topical treatment (for any skin condition, e.g., anti‐cellulite gels or creams)	$223.60	$251.90
Indirect costs		
Base: All who purchase the product	*N* = 117	*N* = 85
Mental health support	$273.00	$440.40
Professional care	$388.50	$698.10
Educational fees	$375.0	$230.50
Healthcare insurance	$483.10	$784.30
Comorbidity costs		
Base: All who purchase the product	*N* = 201	*N* = 187
Medications	$249.60	$301.60
Specialist appointments	$338.40	$456.40
Surgical procedures	$1498.90	$1520.40
Medical tests	$609.50	$326.00
Travel expenses to and from appointments	$346.30	$244.80
Other related costs (e.g., special equipment, non‐surgical procedures)	$644.80	$1146.40
Subtotal: Direct costs	$4090.30	$4524.00
Subtotal: Indirect costs	$1519.60	$2153.30
Subtotal: Comorbidity costs	$3687.30	$3995.70
Total costs (annual)	$9297.20	$10,673.00
Average annual household income	$37,002.62	$53,409.99
Proportion of annual household income spent (%)	25	20

**TABLE 5:** Average yearly expenditure by income level, mean summary (USD).

	Lower income	Middle income	Higher income
Direct costs			
Base: All who purchase the product	*N* = 167	*N* = 143	*N* = 196
Medication	$237.60	$365.50	$477.70
Specialized nutrition + food supplements	$337.10	$326.50	$445.50
Diet regimes	$217.00	$367.50	$706.70
Exercise gear	$322.10	$503.40	$555.20
Medical visits	$281.80	$423.90	$421.70
Consultations with a nutritionist	$338.60	$430.30	$347.50
Specific equipment (e.g., mobility scooter, orthopedics)	$249.40	$197.40	$481.90
Surgical procedures	$184.10	$944.70	$472.90
Residential retreats/wellness camps	$222.40	$141.30	$396.50
Non‐residential retreats/wellness camps	$143.50	$201.10	$333.90
Non‐surgical procedures (e.g., acupuncture, stomach pumps, physiotherapy	$394.70	$354.30	$591.80
Topical treatment (for any skin condition, e.g., anti‐cellulite gels or creams)	$184.30	$214.40	$383.50
Indirect costs			
Base: All who purchase the product	*N* = 74	*N* = 75	*N* = 53
Mental health support	$285.20	$349.30	$412.20
Professional care	$336.60	$355.90	$786.60
Educational fees	$215.60	$307.50	$420.50
Healthcare insurance	$310.80	$398.50	$853.90
Comorbidity costs			
Base: All who purchase the product	*N* = 173	*N* = 127	*N* = 88
Medications	$223.30	$264.00	$382.80
Specialist appointments	$331.90	$327.80	$600.20
Surgical procedures	$1461.80	$2628.0	$834.0
Medical tests	$449.60	$291.10	$672.50
Travel expenses to and from appointments	$226.30	$146.40	$532.90
Other related costs (e.g., special equipment, non‐surgical procedures)	$971.80	$459.10	$963.50
Subtotal: Direct costs	$3112.80	$4470.10	$5614.70
Subtotal: Indirect costs	$1148.10	$1411.20	$2473.10
Subtotal: Comorbidity costs	$3664.80	$4116.40	$3985.90
Total costs (annual)	$7925.70	$9997.70	$12,073.70
Average annual household income	$13,715.26	$32,328.97	$133,030.30
Proportion of annual household income spent (%)	58	31	9

Examining data by BMI class (Table 3), those in BMI Class 2 nearly doubled their expenditures compared to those in BMI Class 1 ($12,437 compared to $4379); although this trend did not hold between BMI Classes 2 and 3, costs were still higher for those in BMI Class 3 than BMI Class 1. Regarding the proportion of household income, BMI Class 2 spent a third (34%) on weight management expenditure, compared to 19% among BMI Class 3, 10% among BMI Class 1%, and 12% among overweight respondents.

Table 4 shows that women spent $1375.80 or 15% more than men overall, spending more on all cost categories by comparison. However, the proportion of annual household income was similar (25% for men, 20% for women) as the average household income was higher for women than men ($53,409 compared to $37,002).

Table 5 shows that OOP expenditures increased by income level, with middle‐ and low‐income respondents spending $2072 more than low‐income respondents (or 26% more), and high‐income respondents spending $4148 more than low‐income respondents (or 52% more). Low‐income respondents were spending on average 58% of their household income on weight management, while middle income respondents were spending 31% and high‐income respondents spent 9%.

Perceived non‐financial costs incurred by PwO are reported in Table 6. Obesity negatively impacted many parts of respondents' personal lives. Half or more of the respondents agreed that obesity affected all the listed aspects of their lives (outside activities, running a household, social life, work, family life, traveling). The ability to do outside activities was most negatively impacted, reported by 74% of respondents overall. Respondents in Spain overall had the lowest agreement that obesity negatively impacted their life. Table 6 also shows respondents' answers to questions on the impact of obesity on their career. Among all respondents, 46% agreed that obesity limited their job prospects, compared to 20% who neither agreed nor disagreed, and 34% who disagreed. Among those who are employed, 46% agreed that due to their excess weight, they were not as productive as they wanted to be; compared to 19% who neither agreed nor disagreed, and 34% who disagreed. 10% of employed respondents reported working from home due to obesity, and 10% reported having to reduce their hours because of living with obesity.

**TABLE 6:** Impact of obesity on life and career, by country.

		Total	Brazil	Spain	Italy	India	Japan	South Korea
*Base*: *All respondents*		*N = 600*	*N = 100*	*N = 100*	*N = 100*	*N = 100*	*N = 100*	*N = 100*
Outside activities (e.g., walking, hiking)	T2B agreement^a^	74% (445)	85% (85)	68% (68)	72% (72)	78% (78)	59% (59)	83% (83%)
	B2B agreement^b^	23% (136)	13% (13)	31% (31)	27% (27)	21% (21)	29% (29)	15% (15)
Running a household (e.g., gardening, housekeeping)	T2B agreement^a^	66% (393)	79% (79)	44% (44)	62% (62)	76% (76)	60% (60)	72% (72)
	B2B agreement^b^	33% (197)	21% (21)	56% (56)	38% (38)	24% (24)	34% (34)	24% (24)
Social life (e.g., meeting with friends, going out)	T2B agreement^a^	62% (369)	75% (75)	40% (40)	55% (55)	76% (76)	53% (53)	70% (70)
	B2B agreement^b^	35% (212)	24% (24)	58% (58)	45% (45)	23% (23)	35% (35)	27% (27)
Work	T2B agreement^a^	59% (355)	74% (74)	34% (34)	48% (48)	72% (72)	53% (53)	74% (74)
	B2B agreement^b^	36% (218)	26% (26)	64% (64)	48% (48)	25% (25)	35% (35)	20% (20)
Family life	T2B agreement^a^	57% (343)	64% (64)	37% (37)	52% (52)	68% (68)	54% (54)	68% (68)
	B2B agreement^b^	41% (247)	35% (35)	63% (63)	47% (47)	31% (31)	41% (41)	30% (30)
Traveling	T2B agreement^a^	55% (331)	67% (67)	31% (31)	49% (49)	67% (67)	48% (48)	69% (69)
	B2B agreement^b^	42% (249)	31% (31)	68% (68)	50% (50)	32% (32)	41% (41)	27% (27)
Obesity limits my career prospects	T2B agreement^c^	46% (275)	56% (56)	32% (32)	44% (44)	58% (58)	44% (44)	41% (41)
	Neither agree nor disagree	20% (119)	17% (17)	18% (18)	19% (19)	17% (17)	23% (23)	25% (25)
	B2B agreement^d^	33% (197)	27% (27)	49% (49)	35% (35)	24% (24)	29% (29)	33% (33)
*Base*: *All respondents who are employed*		*N = 461*	*N = 80*	*N = 72*	*N = 70*	*N = 70*	*N* = 84	*N* = 83
Obesity limits productivity at work	T2B agreement	47% (215)	53% (42)	21% (15)	36% (26)	70% (49)	49% (41)	51% (42)
	Neither agree nor disagree	19% (89)	16% (13)	14% (10)	19% (14)	16% (11)	20% (17)	29% (24)
	B2B agreement	34% (155)	31% (25)	65% (47)	44% (32)	14% (10)	29% (24)	20% (17)
I Am in fear of losing my job due to excess weight	T2B agreement	32% (146)	34% (27)	18% (13)	24% (17)	51% (36)	37% (31)	27% (22)
	Neither agree nor disagree	18% (83)	26% (21)	8% (6)	15% (11)	20% (14)	15% (13)	22% (18)
	B2B agreement	50% (230)	40% (32)	74% (53)	60% (43)	29% (20)	46% (39)	52% (43)

^a^ T2B = Top 2 Box, selecting 1 or 2 on 4‐point Likert scale.

^b^ B2B = Bottom 2 Box, selecting 3 or 4 on 4‐point Likert scale.

^c^ T2B = Top 2 Box, selecting 1 or 2 on 5‐point Likert scale (1: strongly agree; 5: strongly disagree).

^d^ B2B = Bottom 2 Box, selecting 4 or 5 on 5‐point Likert scale (1: Strongly agree; 5: Strongly disagree).

## 4 DISCUSSION

This study provides novel insights into the OOPs accrued by PwO in a wide sample of countries (Brazil, Spain, Italy, India, Japan, and South Korea). It is important to note country health system differences (e.g., quality and coverage of national health insurance programs, and reliance on public vs. private healthcare) will also have a large influence on OOP medical expenses. However, the main finding of this study was that PwO spend a notable amount of their income on OOP expenditures related to weight management, on average, $7351 per year. This accounted for nearly 17% of the annual household income of respondents included in our survey, ranging from 14.1% in Brazil and Spain, up to 24.8% in Japan, which represents a significant financial burden for PwO.

Our research also clearly demonstrates the disproportionate burden that weight management expenditures pose on those with lower socioeconomic status. In our dataset, this was also of particular concern as BMI Class 2 had the highest expenditure, and lowest household income. As BMI has been shown to be linked to lower socio‐economic status^22^, ^23^, ^24^, ^25^ these findings taken together are indicative of a vicious cycle in which overweight and obesity lead to lower socioeconomic status, and obese people with low socioeconomic status face higher OOP expenditures on weight management. In our survey, on average, low ‐ income respondents spent 58% of their annual household income on weight management, placing them well over the threshold for catastrophic health spending. Middle income respondents were also at risk of reaching catastrophic health spending as their weight management expenditure accounted for 31% of household income (compared to 9% of high‐income respondents). These findings are of critical importance given the known risk high OOPs pose to pushing vulnerable people into poverty, combined with the steadily rising burden of obesity and a global cost of living crisis.

There is a clear need to ensure that all PwO and overweight, but particularly those most vulnerable, can access weight management and treatment through means beyond OOP spending—this is a necessary step to achieving the third Sustainable Development Goal of achieving universal health coverage for all.^26^ In 2022, the WHO called for policymakers to ensure reductions in OOP expenditure, particularly for the most vulnerable, by reducing payments proportionate to income.^27^ A 2021 systematic review identified four primary strategies for reducing OOPs in the health system which should be explored in the context of overweight and obesity management: stewardship (e.g., imposing legislation which would strengthen rules and regulations relating to care subsidies and payments), expansion of service delivery, creation of resources and financing mechanisms^2^. Financing mechanisms could include imposing a maximum amount of OOP expenditure in a year for weight management or treatments, or utilizing fixed co‐payments.^27^These options all require significant increased attention and resource allocation within health systems to make a reality.

Our data also show that the burden of OOPs related to obesity is higher for those in BMI classes 2 and 3 compared to BMI Class 1, although the relationship is not linear: in our dataset itemized costs were highest in BMI Class 2. While BMI Class definitions vary by country, our research indicates an urgent need to focus resources on those with BMI >30. This finding mirrors what has been identified in previous research conducted in the US that Obesity classes 2 and 3 were main factors driving spending increases and should be the focus of policies controlling spending, including prevention.^28^

Our findings also demonstrate the burden of direct, indirect and comorbidity related costs: direct costs accounted for 59% of spending on our study, while 24% of costs were indirect, and 17% were spent on comorbidities. These findings differ from a previous study of the societal economic impacts of obesity, which estimated that 36% of costs are direct (of which 91% were medical, 9% were non‐medical) and 64% were indirect (74% premature mortality, 26% productivity losses).^29^ However, the estimates in this study were derived using modeling based on databases (e.g., WHO Global Health Expenditure Database, OECD Health Policy studies, non‐communicable diseases Risk Factor Collaboration, Global Burden of Disease Study, United Nations Population Division, World Bank DataBank) rather than self‐reported data and used a societal economic perspective, leading to the differences in estimated spending and financial impact. Further research is needed to validate these findings on spending related obesity globally. Understanding what drives high individual spending on obesity is also important for understanding the potential costs of obesity treatment and/or prevention programs as well as what can be covered under healthcare insurance policies. Guidelines for the treatment of obesity in the US,^30^ Canada^31^ and Europe^15^ recommend exercise and nutrition as primary treatment for overweight and obesity. However, in our study, high direct costs were driven by spending on surgical and non‐surgical procedures, as well as medical visits, exercise, and diet. Indirect costs were driven by health insurance, and costs related to comorbidities were driven using surgical procedures.

In addition to the OOPs incurred by PwO, our study found that obesity has a negative impact on many parts of peoples' lives. It is already well documented in systematic reviews that individuals who are living with overweight or obese are likely to experience or perceive weight stigma and psychological distress.^32^, ^33^ Our research found that living with obesity impacts productivity at work and depletes individuals' sense of job security because they fear they may lose their employment due to excess weight. This has also previously been found in the literature as evidence from self‐report data, surveys and laboratory research showing that obesity is a general barrier to employment, certain professions and professional successes.^34^Some experimental research has found that hiring discrimination against women living with in particular is statistically and economically significant compared to non‐obese women.^35^ This is a critically important part of the experience of living with obesity, which should be elevated in the public, academic and policymakers' minds given the significant economic burden faced by PwO.

There are several limitations to this research. Self‐reported practices and knowledge are subject to recall and social desirability bias. Our sample may be biased toward those who are willing to pay for interventions, as one of the screening criteria was being willing to pay for a medication for weight loss/management; another exclusion was living in a rural area for this same point to ensure that respondents had sufficient access to weight loss and/or lifestyle modifications. However, the research was designed to intentionally capture respondents who were spending money on managing their overweight or obesity. These results may not be generalizable outside of the countries in which the research was conducted. Finally, this study did not include a weight loss intervention, so there is no way to demonstrate a causal relationship between weight loss and obesity associated costs; as a result, it cannot comprehensively be concluded that reduction in weight completely removes obesity‐related costs. Another potential limitation is that mean expenditure figures are presented; however, due to skewness of expenditure analyzing median figures may have shown differing results. Despite these limitations, there is no comparable research which collects OOP expenditures incurred by PwO in the countries surveyed. This study provides unique and valuable insight into the individual OOP financial burden of living with obesity, with trends in attitudes and spend seen across different countries using a diverse sample. This is an important issue as the number of PwO continues to grow.

## 5 CONCLUSION

This study provides important insight on the individual costs of living with obesity, and demonstrates the significant financial burden that OOPs related to overweight and obesity management pose on people around the world, and in particular the financial risk it poses for those who are most socioeconomically vulnerable. Quantifying the individual economic burden as well as the societal burden of living with obesity can help inform understanding of the economic and political resources required to treat obesity as a disease, which is a global public health imperative that will have large‐scale health and financial repercussions if not addressed. As the number of PwO continues to grow, there is a need for increased action within the public health systems to prevent obesity and avert high levels of both individual spending and societal‐level medical and economic costs; and to find more effective, and cost‐effective treatments, for those living with this disease.

## CONFLICT OF INTEREST STATEMENT

Karine Ferreira and James Baker‐Knight are employees of Novo Nordisk A/S. Evan Kont, Amira Abdelkhalik and Dominic Jones are employees of Ipsos, which received funding to conduct the research. The authors report no other conflicts of interest in this work.

## APPENDIX A

**TABLE A1:** Income classifications per country.

Country	Lower income class	Middle income class	Higher income class
Brazil (BR Real)^a^	Less than 2999	3000–6999	More than 7000
Spain (Euro)^a^	Less than 1999	2000–3999	More than 4000
India (Rupee)^a^	Less than 24,999	25,000–99,999	More than 100,000
Japan (Yen)^a^	Less than 449,999	450,000–749,999	More than 750,000
South Korea (Won)^a^	Less than 2,499,999	2,500,000–6,999,999	More than 7,000,000
Italy (Euro)^b^	Less than 29,999	30,000–49,999	More than 50,000

^a^ Net Monthly household income.

^b^ Gross Annual household income.
